# The relationship between cyberbullying perpetration/victimization and suicidal ideation in healthy young adults: the indirect effects of positive and negative psychotic experiences

**DOI:** 10.1186/s12888-024-05552-2

**Published:** 2024-02-14

**Authors:** Feten Fekih-Romdhane, Diana Malaeb, Nour Farah, Manel Stambouli, Majda Cheour, Sahar Obeid, Souheil Hallit

**Affiliations:** 1 The Tunisian Center of Early Intervention in Psychosis, Department of Psychiatry “Ibn Omrane”, Razi hospital, 2010 Manouba, Tunisia; 2 Faculty of Medicine of Tunis, Tunis El Manar University, Tunis, Tunisia; 3 College of Pharmacy, Gulf Medical University, Ajman, United Arab Emirates; 4 Faculty of Science, Lebanese University, Fanar, Lebanon; 5 School of Arts and Sciences, Social and Education Sciences Department, Lebanese American University, Jbeil, Lebanon; 6 School of Medicine and Medical Sciences, Holy Spirit University of Kaslik, Jounieh, P.O. Box 446, Lebanon; 7 Psychology Department, College of Humanities, Effat University, 21478 Jeddah, Saudi Arabia; 8 Applied Science Research Center, Applied Science Private University, Amman, Jordan

**Keywords:** Cyberbullying, Cyber-victimization, Suicidal ideation, Psychotic experiences, Young adults, Psychosis, Suicide

## Abstract

**Background:**

Even though not all cyber bullies or victims think of (or consider) suicide, they clearly appear to be at an increased risk. One possible strategy to reduce suicide risk is to decrease cyberbullying occurrence; but this approach has its limitations, as it is certainly an illusion to believe that cyberbullying could be controlled or eliminated in a digitalized world. Another alternative and interesting strategy is to consider mediating factors that may indirectly affect suicidality. To this end, our purpose was to test the hypothesis that positive and negative psychotic experiences (PEs) mediate the relationship from cyberbullying perpetration/victimization to suicidal ideation (SI).

**Method:**

The study followed a cross-sectional design, and was conducted during the period from June to September 2022. A total of 3103 healthy community participants from Lebanon were included (mean age 21.73 ± 3.80 years, 63.6% females).

**Results:**

After adjusting over potential confounders, mediation analysis models showed that both positive and negative PEs partially mediated the associations between cyberbullying victimization/perpetration and SI. Higher cyberbullying perpetration and victimization were significantly associated with greater positive and negative PEs; more severe positive and negative PEs were significantly associated with higher levels of SI. Higher cyberbullying victimization and perpetration were significantly and directly associated with higher levels of SI.

**Conclusion:**

In light of our preliminary findings, there appears to be an urgent need for a new focus on carefully assessing and addressing attenuated psychotic symptoms in healthy individuals engaged in cyberbullying either as victims or bullies and who present with SI. It is important that school counselors and decision-makers consider a holistic approach taking into account both external/environmental (bullying) and internal/individual (PEs) factors in their suicide prevention programs. Future longitudinal research in larger samples are still required to confirm our findings and further elucidate the mechanisms underlying the relationship between cyberbullying and suicide.

## Introduction

Suicide is a challenging public health problem around the world. In spite of major prevention efforts of clinicians, researchers, and stakeholders, suicide deaths are still highly prevalent [1]. Based on World Health Organization estimates, 4% of global deaths were from suicide in 2017, with a global mortality rate of 16 per 100,000 each year [2]. What is more concerning is that suicide is consistently found to be the second leading cause of death among the 15- to 29-year-olds [2, 3]. A meta-analysis by Mortier et al. [4] found a lifetime and a 12-month pooled prevalence of suicidal ideation (SI) among college students of 22.3% and 10.6%, respectively. SI often precedes suicide attempt, with over a third of adolescents with SI going on to attempt to take their lives [5]. Despite this large evidence-base, there is limited data regarding population groups that are at high risk for SI [6]. SI has a complex causality, with a broad range of risk factors leading to their onset [7]. In this study, we focused on one of the previously identified risk factors, cyberbullying.

## The relationship between cyberbullying and SI

Cyberbullying perpetration can be defined as an “aggressive, intentional act carried out by a group or individual, using electronic forms of contact, repeatedly and over time against a victim who cannot easily defend him or herself” ( [8], p. 376). Cyberbullying perpetration is generally inflicted through the use of electronic means such as cell phones, computers, and other electronic devices; and is highly prevalent among adolescents and young adults [9, 10]. On the other hand, cyberbullying victimization (also called electronic or Internet victimization) refers to willful and repeated harassment (such as threats, exclusion, humiliation, nasty comments) inflicted to a victim via communication and information technologies [11–13]. The prevalence rate of cyberbullying has significantly increased over the last years in parallel with the increase in the use of technologies in terms of mobile phones, social networking web sites, and internet communities [14]. Meta-analytic estimates revealed that from 2.2 to 56.2% of adolescents reported having been cyber-victimized, while 5.3–31.5% reported having perpetrated cyberbullying [15]. Although both traditional and cyber forms of bullying are harmful, there are concerns that cyberbullying might even be more detrimental due to the fast, easy and repetitive transmission of the harassing behaviors online by perpetrators who are often anonymous, likely to affect multiple victims, and even engage others in harassment [16]. Cyberbullying has major repercussions and long-term devastating effects on youth mental health in both bullies and victims, making it a significant public health concern [17–19]. It is documented in several previous studies that cyberbullying is associated with somatic problems [20], anxiety [21], decreased well-being [22] and self-esteem [23], depression, substance abuse [24, 25], risk behaviors [26], as well as a heightened risk of suicidal ideation and behaviors [24, 25, 27–29].

Multiple cross-sectional [25, 29–33] and longitudinal [34–36] studies showed that cyberbullying is closely related to increased risk of suicidal thoughts and behaviors. Overall, studies found that cyber-perpetrators were more likely to report SI [32, 33] and engage in suicidal behavior [32, 37, 38]. Similarly, cyber-victims were found to be prone to SI [34–36] and suicide attempts [32]. For instance, Hinduja and Patchin [32] found that middle-school students who bullied others online were 1.5 times more likely, and those who were victimized online were 1.9 times more likely, to report suicide attempts compared with those not involved in cyberbullying. A recent longitudinal study revealed that adolescents involved in bullying perpetration were at more than twice the risk of SI (OR = 2.04) and attempted suicide (OR = 2.64) in the following year compared with their noninvolved counterparts [39]. Another three-year cohort study reported that Indian adolescents and young adults who experienced cyber-victimization were are at 2.50-fold increased risk of having SI than those with not cyber-victimized [40].

Despite all this strong evidence, the nature and mechanisms of the relationship between experiencing cyberbullying, either as a victim or as a perpetrator, and suicidality remains largely unclear [41]. Recent growing efforts have been directed towards understanding potential mediators of these relationships [42]. Some previous studies have investigated the mediating effects of a number of mental health factors on the association between bullying perpetration/victimization and suicidal ideation/attempts, including depression [24, 38, 43, 44], perceived stress [24, 45], negative emotions [46, 47], wellbeing [48], satisfaction with life [49], lifestyles [50], and violent behavior [51]. Recently, a systematic review involving 66 studies concluded that factors found to mediate/moderate the association between cyberbullying and suicidality are consistent with those identified in traditional bullying research, which suggests that the mechanisms underlying suicidality appear to involve similar factors in cyberbullying and bullying [52]. In this line of thinking, many factors previously linked to either suicide or cyberbullying have not yet been investigated [53], while they might advance our knowledge and inspire prevention and intervention strategies targeted at reducing youth suicide. In our study, we focused on one of the promising factors that could play a role in the pathway between cyberbullying and SI, psychotic experiences (PEs).

### The hypothesized indirect effect of cyber bullying/victimization on SI through PEs

Through the present study, we intended to add to the body of knowledge by investigating PEs as a theoretically-based mediator in the path from cyber bullying/victimization to SI. This hypothesized model was driven from previous observations that cyber bullying/victimization have been demonstrated to correlate with PEs, which, in turn, were found to lead to SI. Indeed, a recent growing amount of research showed that involvement in cyberbullying either as a victim or as a bully is significantly associated with psychosis. For example, Paruk et al. [54] surveyed South African adolescents aged 13–18 years, and reported that schizophrenia was the second psychiatric disorder most frequently linked to cyberbullying (57.1%) after major depressive disorder (72.4%). Magaud et al. [55] were the first to draw attention to the relatively high prevalence of cyberbullying (38%; mostly through Facebook, text messages, and instant messaging) in individuals at clinical high risk (CHR) for psychosis. Studies in non-clinical populations found similar results. For instance, a cross-sectional study demonstrated that Turkish undergraduate students who were engaged in either cyberbullying victimization or perpetration displayed significantly more severe psychoticism symptoms [56]. Consistently, a study among Chinese high-school students found that PEs were significantly associated with both cyber-bullying (OR = 3.86) and cyber-victimization (OR = 7.59) [57]. A binational study found that cyber-victimization was associated with PEs through the mediating effects of insomnia and distress [58].

On the other hand, there is sufficient and increasing evidence that PEs are predictors of subsequent SI. For instance, a Swedish cohort study reported that adolescents aged 16–17 years who experienced SI and co-occurring PEs had nearly 6 times increased risk of persistence of SI to age 19 to 20 years [59]. Another prospective cohort study across 11 European countries showed that the presence of PEs in adolescents (aged 13–16 years) predicted a nearly 67.50-fold increased risk of acute suicide attempts during the following 12 months [60]. PEs seem to be potential predictors of future suicidal behaviors in individuals with SI [61]; and to increase the risk of SI and suicide attempts, even after accounting for preexisting mental disorders [62] and other common risk factors [63]. A relevant systematic review and meta-analysis in this field documented 2-, 3- and 4-fold increases in odds of later suicide ideation, attempts and death, respectively, in individuals who reported PEs at baseline [64]. Although evidence linking positive PEs to suicidality is well-established, findings on negative PEs are less clear and led to mixed findings; with either positive [65], negative [66, 67], or no significant [68, 69] relationships found between negative symptoms and suicidality. In sum, these data suggest that PEs are markers of suicide risk, which emphasizes their clinical relevance and usefulness in suicide prevention among youth. Potential putative mechanisms underpinning the relationship between PEs and suicidality have been discussed, including shared predisposing genetic and environmental risk factor (such as childhood adversity [61], traumatic brain injury [70], PEs-related psychological distress [71], emotional reactivity to stress [72], as well as nightmares [73–76]).

### The present research

Countries of the Middle East and North Africa (MENA) region are classified as a low- to middle-income countries (LAMIC) [77]. According to the World Health Organization (WHO), an estimated 78% of all suicides occur in LAMIC [1]. In addition, MENA countries have a higher mental disorder burden than the global level [78], and were recognized in 2018 as having one of the highest rates of bullying among adolescents aged 11–15 years [79]. Suicide rates are even suggested to be largely underestimated in Arab countries, where suicide deaths would be often reported as “other violent deaths” [80, 81] due to high stigma and shame directed to both the deceased and their families [82]. Furthermore, the prevalence, nature, impact and response to bullying vary widely across cultures [83]. Despite the fact that cyberbullying has become a widely spread and significant problem in Arab countries among young people [84], only scarce research on this topic has emerged from Middle Eastern countries. The situation in Lebanon is no exception, or even worse, due to the multiple crises the country has been going through over the last years. Studies have, for example, reported high prevalence rates of suicidal ideation (28.9%) among Lebanese adolescents [85] and young adults [86]. Additionally, a study published in 2023 found that 11.3% of Lebanese students were involved in sexual cyberbullying perpetration, and 16.2% of them reported having engaged in embarrassing and inserting malicious content in cyberspace [87]. Even though not all cyber bullies or victims think of (or consider) suicide, they clearly appear to be at an increased risk. One possible strategy to reduce suicide risk is to decrease cyberbullying occurrence; but this approach has its limitations, as it is certainly an illusion to believe that cyberbullying could be controlled or eliminated in a digitalized world. Another alternative and interesting strategy is to consider mediating factors that may indirectly affect suicidality. To this end, our main purpose was to test the hypothesis that PEs mediate the relationship from experiences of cyberbullying perpetration/victimization to SI. Given previous suggestions that negative and positive PEs may be differentially associated with SI, we focused on mediating effects of each PEs dimension separately.

## Method

### Sample and procedure

The present study is part of a large binational, cross-cultural project conducted in Tunisia and Lebanon (The PEARLS [Psychotic Experiences in ARabs from Lebanon and tuniSia] project; *N* = 4891). This project focused, among others, on investigating the nature and correlates of PEs in community young adults of the two countries. The study followed a cross-sectional design, and was conducted during the period from June to September 2022. Participants were deemed eligible if they: (1) were aged 18–35 years (as the at-risk for psychosis population predominantly belongs to this age range [88]), (2) had no self-reported past history of mental illness, including psychosis (previously diagnosed by a mental health provider), (3) had no self-reported history of antipsychotic medication intake, and (4) consented to participate. Participants were excluded if they did not meet the four inclusion criteria. The data was collected through an online survey shared on social media platforms to reach out to as many participants as possible. The present sample involved Lebanese participants only. A total of 4158 participants filled the survey; 1055 were excluded for having self-reported mental health issues; the data of 3103 participants was analyzed consequently. As for ethical considerations, all participants gave their informed consent before inclusion in the study. No compensation or any other form of incentive was provided for study participation. Ethics approval for this project was obtained from the Psychiatric Hospital of the Cross ethics committee (approval code: HPC-013-2022).

### Measures

The first part of the questionnaire contained items about participants’ sociodemographic information, including age, gender, marital status (Married/Single/Divorced/Widowed), educational level (Primary/Secondary/Tertiary), housing area (Urban/Rural), living arrangement (Alone/With family/With friends), substance use (Tobacco/Alcohol/Cannabis/Other drugs), and the presence of any diagnosed psychiatric disorder. The household crowding index and the perceived financial burden were also gathered. The second part comprised four self-report measurement instruments (The Community Assessment of Psychic Experience-42 [CAPE-42], the Columbia Suicide Rating Scale [C-SSRS], and the Revised Cyber Bullying Inventory–II [RCBI-II]).

#### The RCBI-II

This is a 20-item scale composed of two subscales, evaluating either cyberbullying behavior (10 items) or cyber-victimization (10 items) [89]. Items are scored on a four-point scale, ranging from 1 (never) to 4 (more than three times). Higher scores refer to greater cyber-victimization/cyberbullying experiences. These two subscales were separately in analyses. A previously validated Arabic version of the RCBI-II was used [90], and revealed McDonald’s omega coefficients for the cyberbullying and the cyber-victimization subscales of 0.74 and 0.83, respectively, in the current study.

#### The C-SSRS

This scale was designed by investigators in the United States to distinguish the spheres and severity of SI [91]. The original validation study showed that the scale had high reliability, convergent and divergent validity, as well as high specificity and sensitivity for suicidal behavior compared with other SI measures [91]. The scale is composed of 5 questions evaluating SI (i.e., “wish to be dead”, “suicidal thoughts”, “suicidal thoughts with a method”, “suicidal intent”, and “suicidal intent with a specific plan”), which are rated as yes/no. Total scores range from 0 to 5. A score of “0” indicated no SI, and higher scores indicate greater SI. The C-SSRS has been translated into 125 languages, including Arabic, and consistently demonstrated good psychometric properties. It has been is validated in Arabic-speaking adults [101] and adolescents [102] from Lebanon. The present sample yielded a McDonald’s omega of 0.79 for total scores.

#### The CAPE-42

This is a 42-item self-report measure assessing positive, negative and depressive symptoms [92]. The scale involves two dimensions: the first one evaluates the frequency of symptoms on a four-point scale (from 1 = Never to 4 = nearly always), and the second one evaluates the degree of distress related to each experience (from 1 = not distressed to 4 = very distressed). Total scores range from 42 to 168 on both dimensions. Only the positive (20 items) and negative (14 items) subscales have been used in this study. The Arabic validated version of the CAPE-42 was used [93]; which showed excellent psychometric properties. In the present sample, McDonald’s omega value for the positive and negative dimensions were of 0.78 and 0.83, respectively.

### Statistical analysis

SPSS software version 23 was used to conduct data analysis. We had no missing data in our database. McDonald’s omega values were recorded for reliability analysis of all scales and subscales. The Student t test was used to compare two means, whereas the Chi-square test used to compare two categorical variables. Structural equation modeling (SEM) was performed to examine the structural relationship between cyberbullying perpetration/victimization taken as independent variables (X), suicidal ideation as the dependent variable (Y), and positive/negative PE as the mediators (M). The indirect effect was deemed significant if the confidence interval did not pass by zero and if the fit indices of that model were adequate. The fit indices to evaluate the adequacy of the model were the root mean square error of approximation (RMSEA), Goodness of Fit Index (GFI) and the comparative fit index (CFI); RMSEA values ≤ 0.08 or CFI and GFI values > 0.95 indicate a good-fitting model [94]. Results were adjusted over variables that showed a *p* < 0.25 in the bivariate analysis. Significance was set at a *p* < 0.05.

## Results

The mean age of the sample was 21.73 ± 3.80 years (min = 18; max = 35), with 63.6% being females. A total of 584 (18.8%) participants reported SI. Other characteristics of participants are summarized in Table 1.

**Table 1:** Sociodemographic and other characteristics of the participants (*N* = 3103)

Variable	*N* (%)
Gender	
Male	1130 (36.4%)
Female	1973 (63.6%)
Marital status	
Unmarried (Single/Divorced/Widowed)	2800 (90.2%)
Married	303 (9.8%)
Education	
Secondary or less	159 (5.1%)
Tertiary	2944 (94.9%)
Housing area	
Urban	1498 (48.3%)
Rural	1605 (51.7%)
Living arrangement	
Alone	117 (3.8%)
With family	2962 (95.5%)
With friends	24 (0.8%)
Tobacco use	
No	2749 (88.6%)
Yes	354 (11.4%)
Alcohol drinking	
No	2645 (85.2%)
Yes	458 (14.8%)
Cannabis use	
No	3066 (98.8%)
Yes	37 (1.2%)
Other drugs use	
No	3083 (99.4%)
Yes	20 (0.6%)
	**Mean ± SD**
Age (in years)	21.73 ± 3.80
Household crowding index (person/room)	1.51 ± 0.72
Perceived financial burden	5.98 ± 2.64
Suicidal ideation	0.26 ± 0.67
Positive CAPE dimension	31.59 ± 6.27
Negative CAPE dimension	24.29 ± 6.00
Cyberbullying Perpetration	10.99 ± 2.30
Cyberbullying victimization	12.71 ± 3.92

Note. CAPE: Community Assessment of Psychic Experiences

### Bivariate analysis

The results of the bivariate analysis are summarized in Tables 2 and 3. Higher SI was found in females compared to males, and in single participants compared to married ones. Furthermore, higher positive and negative PEs scores, cyberbullying perpetration and victimization, perceived financial burden and household crowding index were significantly associated with more SI.

**Table 2:** Bivariate analysis of factors associated with suicidal ideation (*N* = 3103)

Variable	Mean ± SD	*p*	t	df
Gender		**0.006**	2.730	2442.83
Male	0.22 ± 0.65			
Female	0.28 ± 0.69			
Marital status		**0.005**	2.824	457.19
Single	0.27 ± 0.69			
Married	0.18 ± 0.47			
Education		0.996	0.005	3101
Secondary or less	0.26 ± 0.73			
Tertiary	0.26 ± 0.67			
Housing area		0.330	0.975	3101
Urban	0.27 ± 0.69			
Rural	0.25 ± 0.66			
Living arrangement		0.101	2.298	3100
Alone	0.38 ± 0.93			
With family	0.25 ± 0.66			
With friends	0.13 ± 0.34			
Tobacco use		0.524	0.638	3101
No	0.26 ± 0.66			
Yes	0.28 ± 0.75			
Alcohol drinking		0.224	1.217	3101
No	0.26 ± 0.68			
Yes	0.22 ± 0.65			
Cannabis use		0.548	0.600	3101
No	0.26 ± 0.67			
Yes	0.32 ± 0.91			
Other drugs use		0.152	1.491	19.079
No	0.26 ± 0.67			
Yes	0.65 ± 1.18			

Numbers in bold indicate significant *p* values;

**Table 3:** Correlation of continuous variables with suicidal ideation

	1	2	3	4	5	6	7
1. Suicidal ideation	1						
2. Positive psychotic experiences	0.26***	1					
3. Negative psychotic experiences	0.24***	0.55***	1				
4. Cyberbullying perpetration	0.21***	0.22***	0.13***	1			
5. Cyberbullying victimization	0.25***	0.35***	0.25***	0.45***	1		
6. Age	− 0.02	− 0.01	− 0.01	0.01	− 0.01	1	
7. Household crowding index	0.05**	0.07***	− 0.01	− 0.03	0.003	− 0.17***	1
8. Perceived financial burden	0.04*	0.08***	0.07***	− 0.03	0.001	0.01	0.11***

**p < 0.01; ***p < 0.001; numbers in the table refer to Pearson correlation coefficients

### Mediation analysis

The mediation analysis results were adjusted over the following variables: gender, marital status, living arrangement, alcohol drinking, other illegal drug use, household crowding index, and perceived financial burden. The results of the mediation analysis showed that positive and negative PEs mediated the associations between cyberbullying victimization/perpetration and SI.

**Fig. 1:**
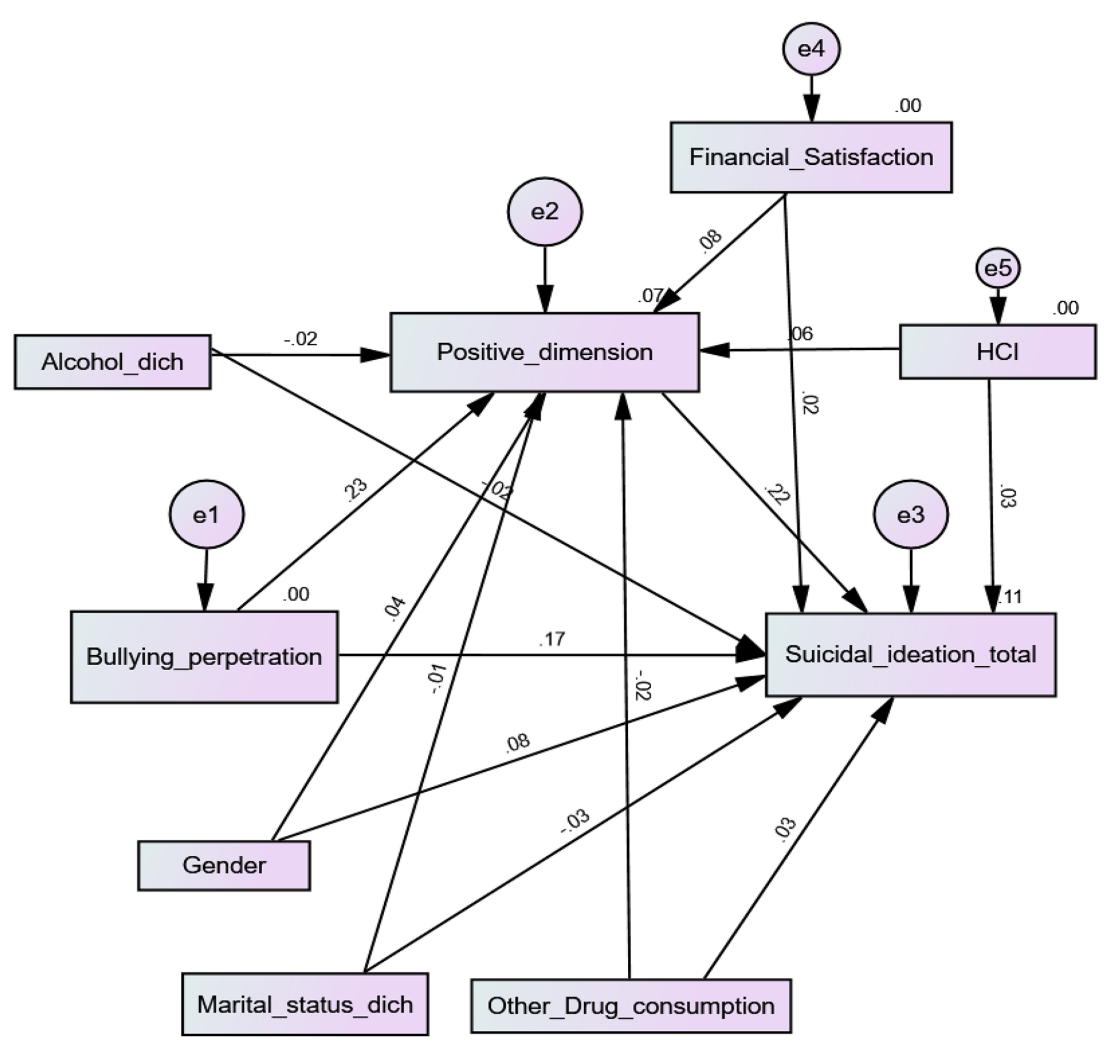
Cyberbullying perpetration, positive psychotic experiences and suicidal ideation interconnections among university students

Higher bullying perpetration was significantly associated with more positive PEs (Beta = 0.23; *p* < 0.001) and more SI (Beta = 0.17; *p* < 0.001). Moreover, more positive PEs was significantly associated with more SI (Beta = 0.22; *p* < 0.001), which shows that positive PEs mediated the association between bullying perpetration and SI (Beta = 0.05; 95% CI 0.03, 0.07; *p* = 0.006) (Fig. 1). The fit indices of this model were adequate for RMSEA = 0.078 (90% CI 0.071, 0.084) and GFI = 0.970 but not CFI = 0.567.

**Fig. 2:**
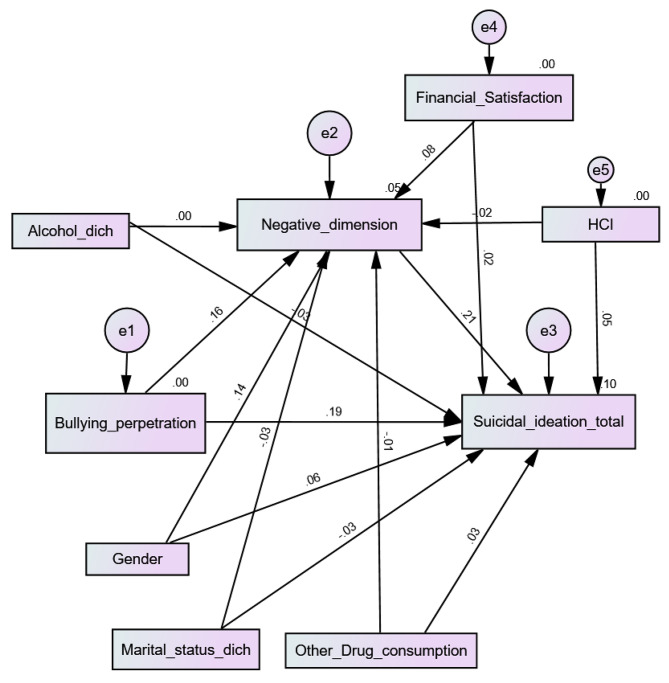
Cyberbullying perpetration, negative psychotic experiences and suicidal ideation interconnections among university students

Higher bullying perpetration was significantly associated with more negative PEs (Beta = 0.16; *p* < 0.001) and more SI (Beta = 0.19; *p* < 0.001). Moreover, more negative PEs were significantly associated with more SI (Beta = 0.21; *p* < 0.001), which shows that negative PEs mediated the association between bullying perpetration and SI (indirect effect: Beta = 0.03; 95% CI 0.02, 0.05; *p* = 0.007) (Fig. 2). The fit indices of this model were adequate for RMSEA = 0.078 (90% CI 0.071, 0.084) and GFI = 0.970 but not CFI = 0.528.

**Fig. 3:**
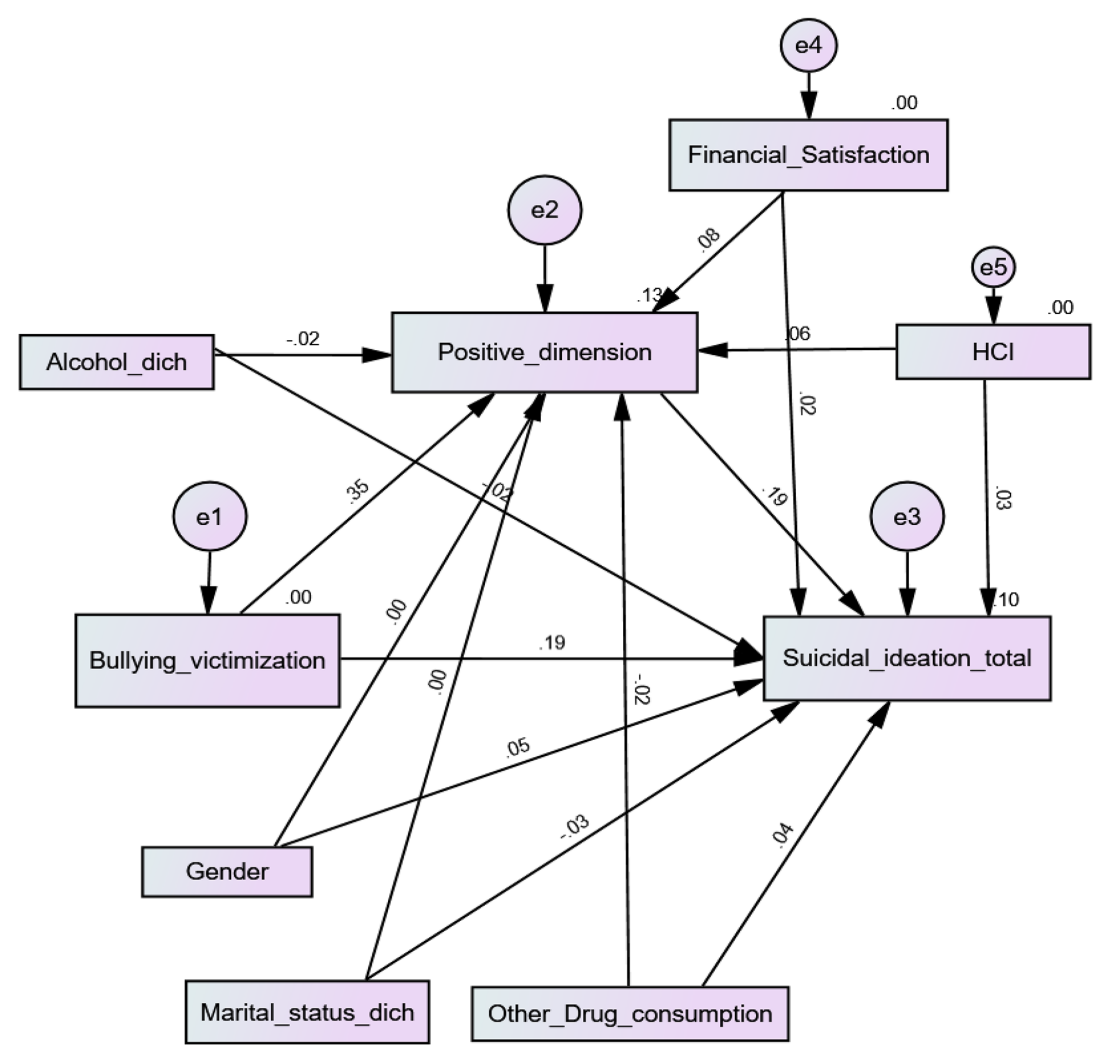
Cyberbullying victimization, positive psychotic experiences and suicidal ideation interconnections among university students

Higher bullying victimization was significantly associated with more positive PEs (Beta = 0.35; *p* < 0.001) and more suicidal ideation (Beta = 0.19; *p* < 0.001). Moreover, more positive PEs were significantly associated with more SI (Beta = 0.19; *p* < 0.001), which shows that positive PEs mediated the association between bullying victimization and SI (Beta = 0.07; 95% CI 0.05, 0.09; *p* = 0.005) (Fig. 3). The fit indices of this model were adequate for RMSEA = 0.066 (90% CI 0.059, 0.073) and GFI = 0.979 but not CFI = 0.731.

**Fig. 4:**
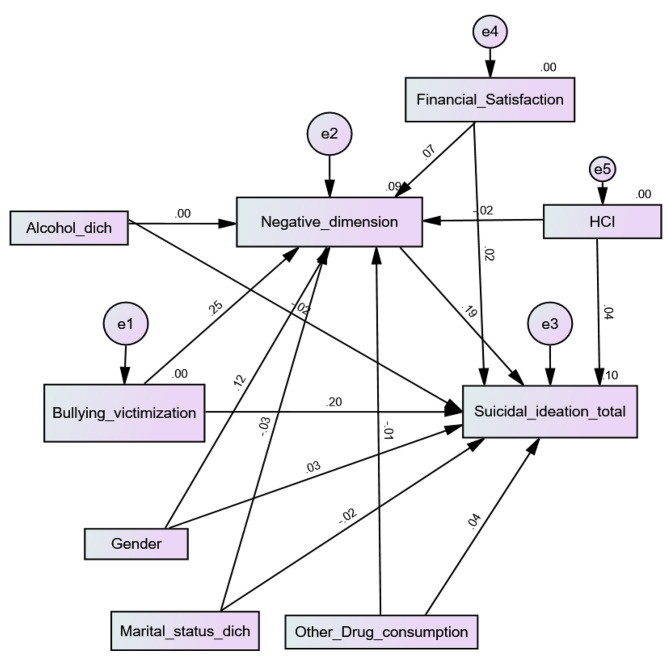
Cyberbullying victimization, negative psychotic experiences and suicidal ideation interconnections among university students

Higher bullying victimization was significantly associated with more negative PEs (Beta = 0.25; *p* < 0.001) and more SI (Beta = 0.21; *p* < 0.001). Moreover, more negative PEs were significantly associated with more SI (Beta = 0.19; *p* < 0.001), which shows that negative PEs mediated the association between bullying victimization and SI (Beta = 0.05; 95% CI 0.03, 0.06; *p* = 0.005) (Fig. 4). The fit indices of this model were adequate for RMSEA = 0.066 (90% CI 0.059, 0.073) and GFI = 0.979 but not CFI = 0.678.

## Discussion

Our understanding is still limited with respect to whether the involvement in cyberbullying perpetration and experience of cyber-victimization are associated with SI, and to the determination of potential mediating factors. To bridge this knowledge gap, we sought to examine the direct and indirect effects of cyberbullying perpetration/victimization on SI via positive PEs. Examining PEs as a mediator can help inform intervention approaches supporting youth mental health. Our hypothesis was partly confirmed, as positive PEs partially mediated the path between both cyber-victimization, cyberbullying and SI. This suggests that both cyberbullying victims and perpetrators may be more at risk of SI when they experience more severe subthreshold positive psychotic symptoms.

With regard to direct effects, both cyberbullying and victimization were positively associated with SI in our sample of young adults, which is consistent with the weight of empirical evidence [32, 33, 46, 95, 96]. Indeed, previous longitudinal studies indicated that youth who are involved in cyberbullying victimization and offending have an increased likelihood of suicidal thought, attempts, and death (e.g., [34–36, 39, 40]). Interestingly, prior research showed that adolescent cyber-perpetrators reported higher SI levels over and beyond being involved in traditional bullying [33]. In addition, prospective evidence pointed to the differential impact of traditional versus cyber forms of bullying on suicide risk. For instance, two studies found that cyber-victimization, but not traditional victimization, was linked to SI [34, 36]; thus supporting that cyberbullying is more harmful on youth mental health than face-to-face bullying. It is worth noting, however, that bidirectional associations between cyberbullying and SI were previously reported [35]. Therefore, our conclusions can only be preliminary because of the cross-sectional design; and future longitudinal research is still required to make assumptions about causality.

Beyond examining the direct link between cyberbullying and SI, previous researchers called for research on explaining mechanisms of this relationship that might be useful in providing more effective targets for intervention [60]. Motivated by this call, we chose to investigate the indirect effects of both positive and negative PEs. Our two mediation models were significant, which is in agreement with previous studies indicating that cyberbullying is associated with PEs [56, 57], and that PEs predict subsequent suicidality [59–64]. This cautiously suggests that being either a cyberbully or a cyber-victim seems to increase the chances of developing PEs, leading in turn to greater suicidal thoughts. These findings are also broadly in line with the observations of Hinduja and Patchin [97] who proposed that, when some factors co-occur with cyberbullying, they constitute an amalgamation of different painful external and internal experiences, and together contribute to the severe outcome of SI. Nevertheless, here again we warn readers that our results are only correlational in nature, and directionality cannot be determined. As previous research empirically demonstrated that the prospective relationship between adversity experiences and psychosis is bidirectional [98], it can also be suggested that psychotic symptoms may precede the occurrence of cyberbullying. In this case, individuals with psychotic symptoms (including paranoia) may be more prone to perceive neutral or benign electronic interactions as negatively directed toward them, or may also use electronic venues to react to, or mitigate their psychotic symptoms. This avenue should be explored in future research. We believe, however, that these observational findings may help in understanding the interplay between cyberbullying, PEs, and SI; and may guide future research and clinical interventions directed at preventing suicide in youth.

### Clinical and research implications

In this study, we investigate, for the first time, the mediating effect of PEs on the relationship cyberbullying in its two facets (perpetration and victimization) and SI. Identifying the proneness to experience subclinical psychotic symptoms as an underlying mediator of this relationship might have important clinical implications. In light of our preliminary findings, there appears to be an urgent need for a new focus on carefully assessing and addressing attenuated psychotic symptoms in healthy individuals engaged in cyberbullying either as victims or bullies and who present with SI. This agrees with the suggestions of Kelleher et al. [60] that assessment of PEs “should be considered a key element of suicide risk assessment”. These researchers even went as far as to suggest that all future research on suicidality should incorporate a measure of psychotic symptoms [60]. Overall, it is important that school counselors and decision-makers consider a holistic approach taking into account both external/environmental (bullying) and internal/individual (PEs) factors in their suicide prevention programs. Multilevel suicide prevention interventions (i.e., combined interventions by different providers in multiple domains including teachers and priests at the community level, general practitioners at the primary care level) that have previously showed effectiveness in other contexts and backgrounds should be widely implemented in Lebanon [99]. Furthermore, mental health providers, psychologists, educators and school administrators need to give necessary attention and concern to cyberbullying among vulnerable young people who present with SI. Effective, evidence-based cyberbullying intervention programs involving communication and social skills, digital citizenship, empathy training, coping skills, as well as education on cyberbullying for both the individual youth and parent can help overcome suicide risk in young people who experience cyberbullying [100].

### Limitations

The present study has certain limitations that merit to be acknowledged. The main limitation stems from the cross-sectional design. Additional longitudinal studies are required before drawing any firm conclusions. In addition, the convenience sampling procedure prevents any possible generalization of our findings. Moreover, since the sample included a majority of females, unmarried, highly educated and residing with family members, the results cannot be generalized to the general public. Furthermore, due to its self-report nature, the study may be subject to social desirability and response bias. Finally, other characteristics of cyberbullying need to be examined in future research, such as type of technology means, technology addiction, parental online supervision and anonymity [52].

## Conclusion

The remaining high rates of suicidality in adolescent and young adult populations despite huge efforts suggests that more effective strategies and approaches are urgently needed, while considering new targets for intervention research and practice. By showing that PEs can significantly potentiate the effect of cyberbullying on SI, we suggest that it may be beneficial to think of tackling psychotic symptoms in clinical and preventive programs for youth at-risk of suicide, who are involved in cyberbullying behavior and/or victimization. This points to the importance of incorporating both individual and environmental factors when developing anti-suicide interventions. Future longitudinal research in larger samples are still required to confirm our findings and further elucidate the mechanisms underlying the relationship between cyberbullying and suicide.

## Data Availability

All data generated or analyzed during this study are not publicly available due the restrictions from the ethics committee. Reasonable requests can be addressed to the corresponding author.
